# Simple application of adipose-derived stem cell-derived extracellular vesicles coating enhances cytocompatibility and osteoinductivity of titanium implant

**DOI:** 10.1093/rb/rbaa038

**Published:** 2020-12-03

**Authors:** Lifeng Chen, Shan Mou, Jinfei Hou, Huimin Fang, Yuyang Zeng, Jiaming Sun, Zhenxing Wang

**Affiliations:** 1 Department of Plastic Surgery, Union Hospital, Tongji Medical College, Huazhong University of Science and Technology, Wuhan 430022, China; 2 Wuhan Clinical Research Center for Superficial Organ Reconstruction, Wuhan Union Hospital, Huazhong University of Science and Technology, 1277 Jiefang Avenue, Wuhan 430022, China

**Keywords:** adipose-derived stem cell-derived extracellular vesicles, titanium surface modification, fibronectin, osteoinductivity

## Abstract

Surface modification using bioactive molecules is frequently performed to improve the biological properties of medical metal biomaterial titanium (Ti) implants. Developmental evidence suggests that mesenchymal stem cell-derived extracellular vesicles (MSC-EVs) served as potent bioactive component. As a subset of MSC-EV, adipose-derived stem cell-derived extracellular vesicles (ADSC-EVs) could be obtained from abundant adipose tissue. Meanwhile, it possesses multiple regenerative properties and might be used to endow biological activities to medical Ti implant. Here, we present a simple ADSC-EV coating strategy based on physisorption of fibronectin. This ADSC-EV functionalized Ti implants (EV-Ti) revealed enhanced osteoblast compatibility and osteoinductive activity. Cell spreading area of EV-Ti group was 1.62- and 1.48-fold larger than that of Ti group after 6 and 12 h of cell seeding, respectively. Moreover, EV-Ti promoted alkaline phosphatase, collagen 1 and osteocalcin gene expression in osteoblast by 1.51-, 1.68- and 1.82-fold compared with pristine Ti, respectively. Thus, the MSC-EVs modification method reported here provide a clinically translatable strategy to promote the bioactivity of Ti implants.

## Introduction

Titanium (Ti) implants and their alloy have been widely used in clinical including arthroplasty, craniofacial surgery and orthodontic implant for decades of years since the inception of dental implants in 1969 [1]. The merits of these medical metal implants are attributed to their biocompatibility and mechanical properties that make them suitable for bone replacement compared with other metallic biomaterials [2]. Moreover, they are corrosion resistance and demonstrate bio-inert behavior [3]. However, the possibility of aseptic loosening, shifting or even detachment has always existed after long-term implanting, which may be caused by insufficient osseointegration of pure Ti [4]. 

Osseointegration, originally introduced by Adell [1] and supplemented by Albrektsson *et al.* [5] indicate ‘a process whereby clinically asymptomatic rigid fixation of alloplastic materials is achieved and maintained in bone during functional loading’. One of the main factors affecting osseointegration is the interaction between materials and the direct contacted cells that are regulated by the morphology, texture and composition of the implants [6]. Typically, bioactive components, including protein, polypeptide and nucleic acid, play an important regulatory role.

Extracellular vesicles, first recognized during maturation of reticulocytes in 1983, are cell-derived membranous nanoparticles secreted by a variety of cell types and body fluids studied to date [7, 8]. A series of researches have shown that mesenchymal stem cell-derived extracellular vesicles (MSC-EVs) could promote tissue repairing and regeneration, as they retain similar features of their sourced cell [9]. Particularly, they could mediate the paracrine effects of their parent cells by transporting enclosed complex molecules including proteins, nucleic acids, lipids and sugars [10]. Taking advantage of some characteristics of the MSC-EV: (i) MSC-EVs have numerous therapeutic potentials mediated by multiple mechanisms; (ii) the membrane of EVs can act as a protective carrier for bioactive signaling molecules; (iii) they can be stored at low temperature (−20°C or −80°C) for a long time without the addition of toxic cryo-preservatives [11, 12]. Adipose-derived stem cells (ADSCs) are a subset of MSCs that can be obtained easily from abundant adipose tissues. Meanwhile, ADSC-EVs possess many regenerative properties, such as anti-inflammatory, angiogenesis and osteoinduction [13, 14]. Therefore, ADSC-EV may serve as an ideal substance for medical mental material biological modification.

Current methods used to load EVs including polyethylenimine coating adsorption [15], hydrogel mixture [16] and avidin−biotin complex combination [17]. However, these methods hold some limitations including potential biosafety, heterogeneous distribution and tedious operation steps. Fibronectin, an extracellular matrix protein found in blood and connective tissues, is involved in functions such as cell adhesion, growth and migration. Therefore, fibronectin coatings have become a primary choice to enhance bioactivity of bioinert material [18]. Previous studies have indicated that fibronectin can be easily adsorbed onto Ti surface to enhance the surface properties, cellular performance and bone integration efficacy [19]. Moreover, our research has indicated that fibronectin can be used to tether EVs onto decalcified bone matrix [20]. In the present work, we fabricated an ADSC-EV coating onto Ti implants which relied on fibronectin as a simple MSC-EV-based medical implant biofunctionalization strategy.

Specifically, ADSC-EVs were extracted by ultra-centrifugation. Then, their morphology and average diameter were characterized by scanning electron microscope (SEM) and nano tracking analysis (NTA). The uptake process of EVs by osteoblast was observed by fluorescent labeling. Then, ADSC-EVs were immobilized onto fibronectin coated Ti and the EVs coated Ti (EV-Ti) was observed by fluorescence microscope and SEM. Cell responding including cellular morphology and osteo-related gene expression was detected by fluorescence staining and real-time quantitative reverse transcription polymerase chain reaction (qRT-PCR).

## Materials and methods

### Isolation, culture and identification of ADSCs

Human fat tissue was obtained from female patients (20–40 years old) who underwent abdominal liposuction in Wuhan Union Hospital and the protocol was approved by the Ethics Committee of Huazhong University of Science and Technology.

HADSCs were isolated according to our previous research [14]. Simply, the liposuction aspirates were digested in 0.1% NB4 collagenase (SERVA, Germany) at 37°C for 2 h and then the suspension was centrifuged at 280 g for 10 min. After which the pellet was resuspended in Dulbecco’s modified Eagle’s medium (DMEM, Thermo Fisher Scientific, USA) mixed with 10% fetal bovine serum (FBS, Thermo Fisher Scientific) and 1% gentamycin/streptomycin (Thermo Fisher Scientific) and then filtered through cell strainer (70 µm, Falcon, USA) to collect single cell suspension that was subsequently seeded in dishes and incubated at 37°C in an atmosphere of 95% air/5% CO_2_.

Immunofluorescent staining was used to identify hADSC phenotype. Antibodies of CD29 (ab134179), CD44 (ab6124), CD90 (ab181469), CD105 (ab11414) and CD45 (ab40763) were purchased from Abcam, Inc. (USA). The immunophenotype of hADSC was detected using corresponding primary antibodies above, followed by corresponding IgG conjugated with FITC. The results were observed with upright fluorescence microscope (Ni-E, Nikon, Japan).

Adipogenesis, osteogenesis and chondrogenesis differentiation potential of hADSCs were assessed. For adipogenesis identification, hADSCs were cultured in addition of 0.5 mM 3-isobutyl-1-methylxanthine, 10 μM insulin, 200 μM indomethacin and 1 μM dexamethasone for 3 weeks and were then incubated in 0.5% (wt/vol) oil red O for 30 min. For osteogenesis identification, hADSCs were cultured in addition of 10 mM β-glycerophosphate, 50 μM ADSCorbic acid and 0.1 μM dexamethasone for 2 weeks and were then stained with Alizarin Red. For chondrogenesis identification, hADSCs were incubated in addition of 1 mM sodium pyruvate, 1% insulin-transferrin sodium-selenite, 0.17 mM ADSCorbic acid, 0.35 mM l-proline, 1.25 mg/ml bovine serum albumin, 5.33 μg/ml linoleic acid, 0.1 μM dexamethasone and 0.01 μg/ml transforming growth factor-β for 4 weeks and they were then assessed by Toluidine Blue staining. All results were observed with upright microscope (Ni-E, Nikon).

### Separation and characteristics of hADSC-EVs

To hADSC-EVs isolation, continuous centrifuge was performed. Briefly, the culture medium of hADSCs (4 − 6 passage) was changed with serum-free DMEM medium for 24 h. Then, the solution was centrifuged at 2000 g for 20 min at 4°C to discard large debris. Finally, the EVs was separated by further centrifugation at 16 000 g for 1 h at 4°C. The protein concentration of EVs was determined using BCA Protein Assay kit (Thermo Fisher, USA). The size distribution of EVs was assessed by Nanosight (NS300, Malvern, UK) at room temperature.

The morphology of hADSC-EVs was characterized using transmission electron microscope (TEM, Hitachi, Japan). Specifically, hADSC-EV samples were seeded on a formvar-carbon-coated grid and then fixed with 1% glutaraldehyde for 5 min followed by staining with 2% uranyl acetate. After drying at room temperature, the sample was observed with an HT7700 microscope.

To observe hADSC-EV under fluorescent, PKH26 (Merck Life Science, China) staining was applied. Specifically, hADSC-EVs (100 µg) were first centrifuged at 16 000 g for 1 h at 4°C and then re-suspended in diluent C (1 ml) mixed with PKH26 solution (5 µl). After incubating for 30 min at 37°C, the liquor was removed by centrifuging at 16 000 g for 1 h at 4°C. The labeled EVs pellet was resuspended in PBS and observed under laser scanning confocal microscope (CLSM, Nikon).

### HADSC-EV uptake assay

The PKH-26 labeled hADSC-EVs mentioned above were used to determine their uptake by human osteoblast cell line MG63 (Cell Bank, China). Specifically, labeled hADSC-EVs (1 µg/µl, suspended in PBS) were incubated with MG63 for 0.5, 4 and 8 h, respectively. Following incubation, cells were washed with PBS twice, then fixed in 4% paraformaldehyde for 10 min and washed again. After staining with 6-diamidino-2-phenylindole (DAPI) for nuclei, all samples were captured under upright fluorescence microscope (Ni-E, Nikon). The hADSC-EV uptake rate was calculated by the following formula:

$$\text{Uptake}\;\text{ratio}\;\left(\%\right)=\frac{U}{T}\times 100\%$$

where ‘*U*’ represents the number of cells that have uptake EVs (staining with both nuclei and PKH26), ‘*T*’ represents the number of total cells, five fields were randomly selected for calculation.

### Preparation and observation of EV-Ti

Pure Ti (BAOTI Group Co, China, 5 mm diameter) substrates were polished with SiC sandpaper of 280, 400, 800, 1000 and 1200 # successively and then ultrasonically cleaned with isopropyl alcohol, absolute ethanol and deionized water for 10 min. After dried in air, all the samples were sterilized with ethylene oxide and then soaked in fibronectin solution (10 μg/ml) with shaking at 37°C for 12 h. To fabricated EV-Ti, hADSC-EVs were dropped onto prepared Ti substrate (10 μg for each) and incubated at 37°C for 2 h.

### Cell compatibility of EV-Ti

To evaluate the cellular compatibility of EV-Ti, MG63 was seeded onto Ti and EV-Ti with the initial density of 5 × 10^4^/ml and cultivated in Minimum Eagle’s Medium-Alpha (MEM-α, Hyclone, China) containing 10% FBS and 1% penicillin/streptomycin. After 6 and 12 h of culture, all the samples were washed with PBS and then fixed with 4% paraformaldehyde. The F-actin and nucleus of cells on each substrate were stained using tetramethylrhodamine conjugate phalloidin (AAT Bioquest, USA) and DAPI, respectively. Analysis of cell morphology was conducted using upright fluorescence microscope (Ni-E, Nikon). Cellular spread area and perimeter were analyzed with ImageJ, five fields were randomly selected for calculation.

### Osteoinduction of EV-Ti

To evaluate the *in vitro* osteoinduction ability of EV-Ti, the expression of osteogenesis-related proteins and genes in osteoblast cultured on different materials were evaluated. After 14 days of culture (with the cell initial density of 5 × 10^4^/ml), all samples were fixed with paraformaldehyde (4%), permeabilized with Triton X-100 (0.1%), and blocked with bovine serum albumin. Then the treated samples were incubated in primary antibody against collagen 1 (ab34710, Abcam, Inc., 1:200 dilution) or osteocalcin (Proteintech Group, Inc., China, 1:200 dilution) at 4°C with shaking overnight, followed by incubating in specified secondary antibodies (Abcam, Inc., 1:500 dilution) for 2 h. Cell nucleus were counterstained with DAPI. Results were observed with upright fluorescence microscope (Ni-E, Nikon). The mean integrated option density (IOD) value of each group was quantified using ImageJ.

qRT-PCR was used to analyze osteogenesis-related gene expression. Osteoblasts were seeded onto Ti and EV-Ti with the initial density of 5 × 10^4^/ml. After 14 days, total RNA of cells was isolated using TRIpure reagent and then reverse transcribed into complementary DNA using Revertaid reverse transcriptase according to the manufacturer’s instruction. Finally, qRT-PCR was conducted using KAPA SYBR FAST qPCR Kit (KAPA, China) on the StepOnePlus Real-Time PCR System (Applied Biosystems, USA). Relative expression level of each gene (ALP, COL 1 or OCN) was normalized to the housekeeping gene β-actin. The primer sequences were as follows: ALP (F: ACCACCACGAGAGTGAACCA, R: CGTTGTCTGAGTACCAGTCCC); COL 1 (F: GAGGGCCAAGACGAAGACATC, R: TCTCAGATCGTTGAACCTTGCTA); OCN (F: ACCACATCGGCTTTCAGGA, R: CCATAGGGCTGGGAGGTCA).

### Statistical analysis

Parametric data were presented as mean ± standard deviation (SD) and comparisons among different groups were assessed by unpaired *t*-test or one-way ANOVA test. The significance level was labeled by asterisks (**), referring *P* < 0.01.

## Results

### Characteristics of hADSC-EVs

**Figure 1.**
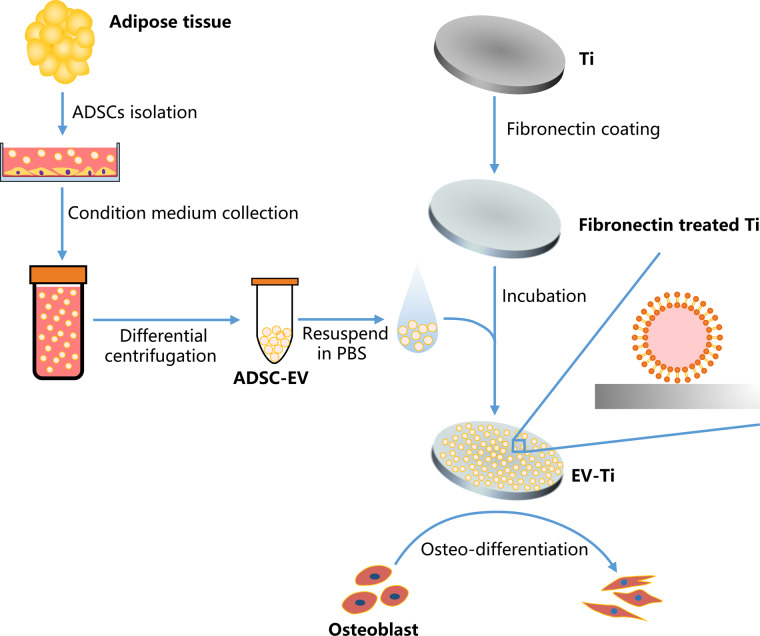
Schematic representation of the entire experimental process. ADSC-EVs were separated from ADSCs condition medium after differential centrifugation and then incubated onto fibronectin treated titanium to form EV-Ti. Next, the cellular compatibility and osteoinductive ability of EV-Ti was evaluated by observing the osteoblast cultured on the substrates

**Figure 2.**
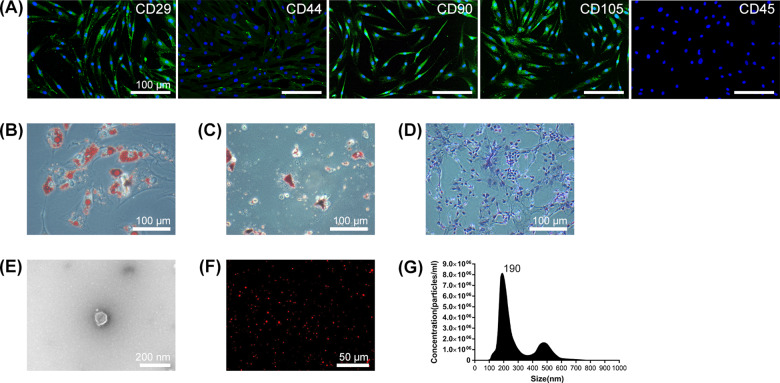
Characteristics of hADSC and hADSC-EVs. (**A**) Representative fluorescent images indicating positive expression of mesenchymal markers (CD90 and CD105) and cell adhesion molecules (CD29 and CD44), as well as negative expression of hematopoietic marker CD45. Green: corresponding immunophenotype markers; blue: DAPI; scale bar: 100 μm. Lipid vacuole, extracellular calcium deposition and glycosaminoglycan of hADSC can be observed after adipogenic, osteogenic and chondrogenic induction, as representative image shown in (**B**), (**C**) and (**D**), respectively. Scale bar: 100 μm. (**E**) TEM observation confirming lipid-bilayered structure of hADSC-EVs. Scale bar: 200 nm. (**F**) CLSM image showing positive expression of phospholipid membrane probe. Red: PKH26; scale bar: 50 μm. (**G**) NTA showing that the largest average hydrodynamic diameter of hADSC-EVs is 190 nm

**Figure 3.**
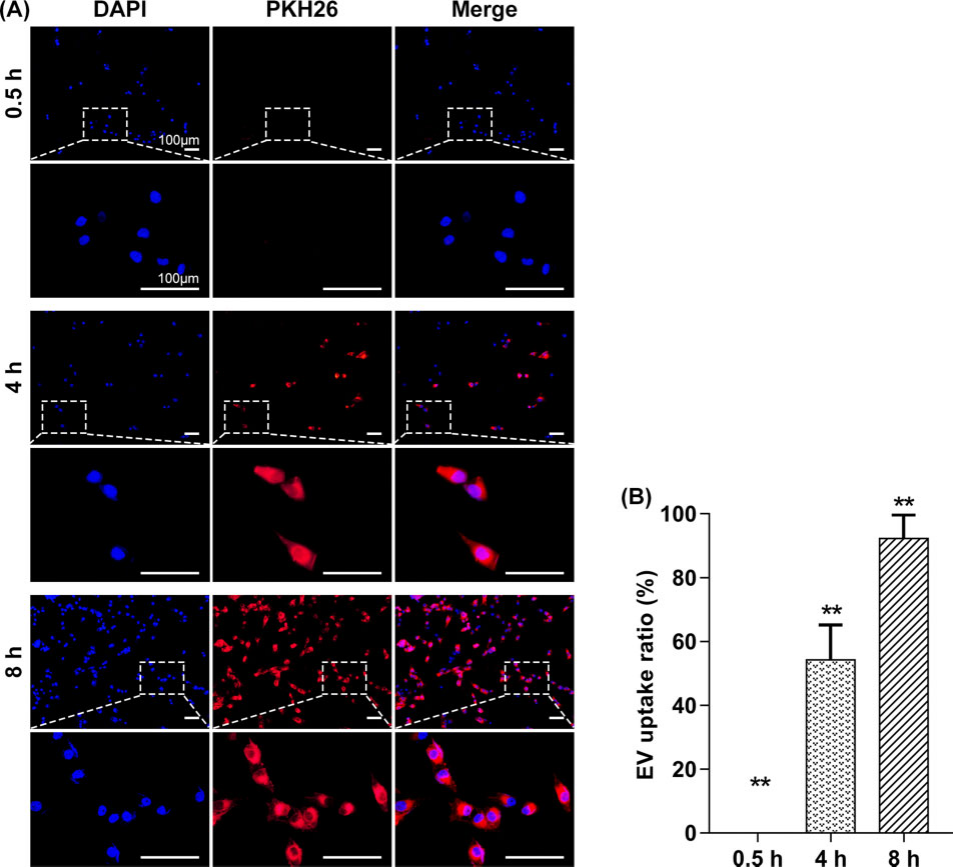
(**A**) Representative images showing that the amounts of osteoblasts which uptake hADSC-EVs gradually increase with the extension of incubation and almost all cells have uptake EVs at 8 h. Red: PKH26; blue: DAPI; scale bar: 100 μm. (**B**) Quantitative result of EV uptake ratio is consistent with the above finding (*N* = 3, asterisk indicates significant difference, *P* < 0.01 by one-way ANOVA test)

**Figure 4.**
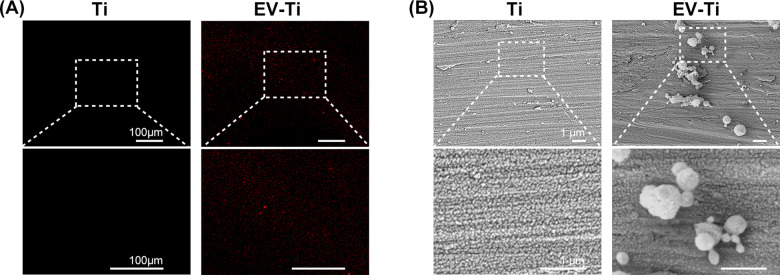
Observation of Ti and EV-Ti. (**A**) Positive fluorescent expression of hADSC-EVs can be observed under EV-Ti compared with unmodified Ti. Red: PKH26; scale bar: 100 μm. (**B**) SEM images showing that hADSC-EVs are distributed on the surface of EV-Ti. Scale bar: 1 μm

**Figure 5.**
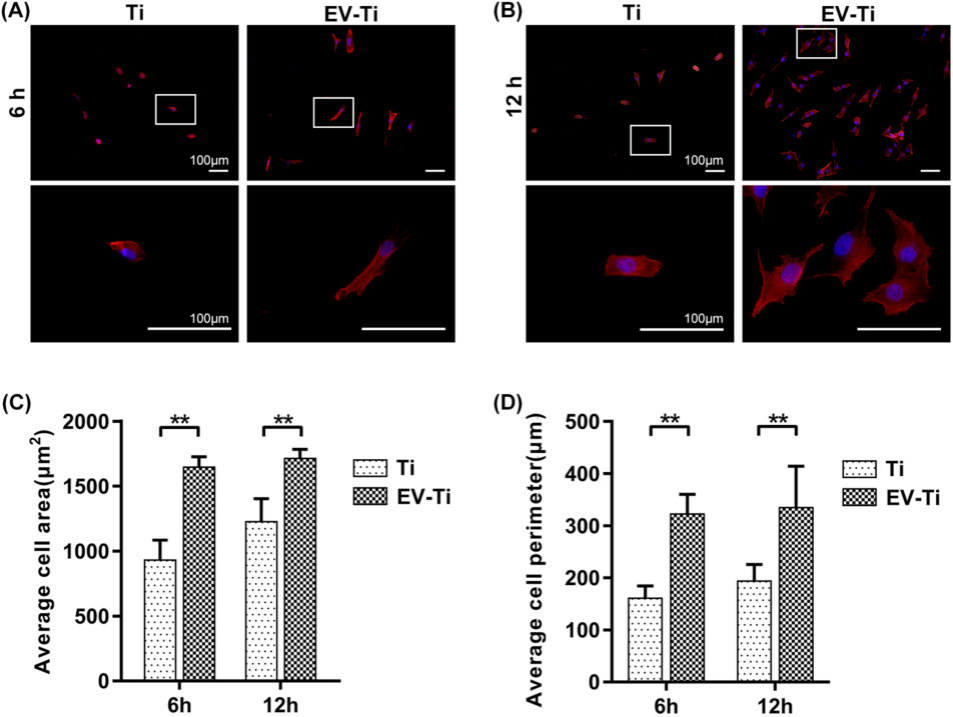
Cell compatibility of EV-Ti. Representative fluorescent images showing that osteoblast cultured on EV-Ti have larger projected area and affluent pseudopods compared with that cultured on Ti after (**A**) 6 h and (**B**) 12 h incubation. Red: phalloidin; blue: DAPI; scale bar: 100 μm. White square indicates high-magnitude field of specific field. Quantitative analysis of cells’ (**C**) projected area and (**D**) perimeter on Ti, and EV-Ti groups matching the results above (*N* = 3, asterisk indicates significant difference, *P* < 0.01 by Student’s *t*-test)

**Figure 6.**
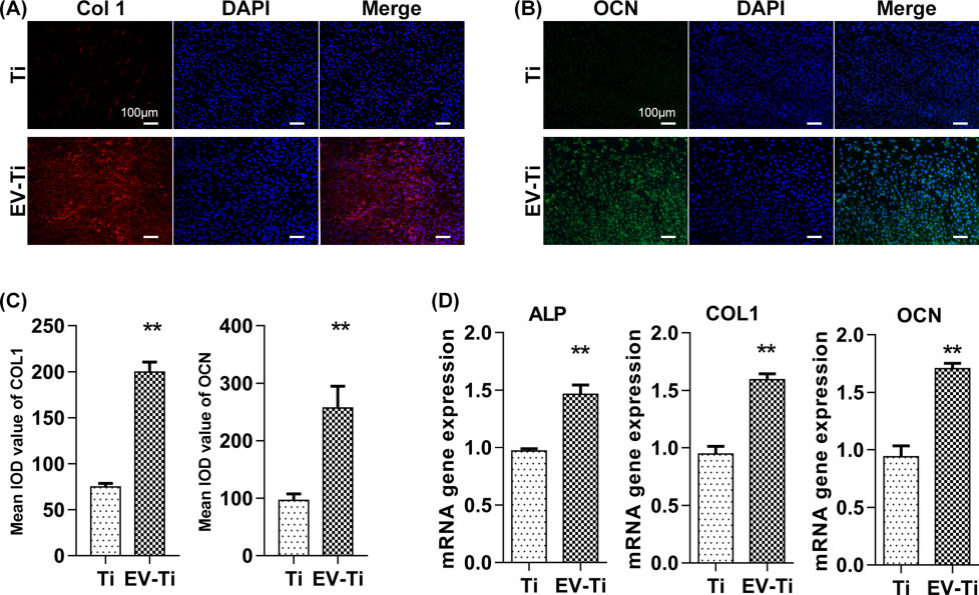
Osteoinductive ability of EV-Ti *in vitro*. Osteoblast was cultured on Ti and EV-Ti for 14 days and then osteoinductive-related genes proteins or genes were evaluated. Immunofluorescence images showed in (**A**) and (**B**) revealed that the osteoblasts seeded on EV-Ti had higher expression of COL 1 and OCN compared with that seeded on Ti, respectively. Red: COL 1; green: OCN; blue: DAPI; scale bar: 100 μm. (**C**) Quantitative analysis of integrated option density (IOD) estimated by ImageJ (*N* = 3, asterisk indicates significant difference, *P* < 0.01 by Student’s *t*-test). (**D**) Expression of ALP, COL 1 and OCN genes in osteoblast cultured on EV-Ti were significantly higher compared with those cultured on Ti (*N* = 3, asterisk indicates significant difference compared with Ti group, *P* < 0.01 by Student’s *t*-test)

The entire process of experiment was schematic represented in Fig. 1. Specifically, hADSCs were isolated from human adipose tissue, and hADSC-EVs were separated from the condition medium of hADSCs. The immunophenotype and differentiation capability of hADSCs and morphology, size distribution and cellular internalization of hADSC-EVs were characterized and showed in Figs 2 and 3. The EV-Ti was fabricated by incubating hADSC-EV onto the surface of Ti overnight. The immobilization of hADSC-EVs was observed and presented in Fig. 4. Then, cell compatibility and osteoinduction of this modified implant were observed and evaluated in Figs 5 and 6.

As shown in Fig. 2A, the extracted hADSCs were positive expression of CD29 and CD44, which present cell adhesion molecules, and CD90 and CD105, which present mesenchymal markers. And the hematopoietic marker CD45 was negative observed. The lipid vacuole can be detected by Oil Red O staining after adipogenic induction for 3 weeks (Fig. 2B), as well as calcium nodules by Alizarin Red staining following osteogenic induction for 2 weeks (Fig. 2C) and glycosaminoglycan by Toluidine Blue staining since chondrogenic induction for 4 weeks (Fig. 2D).

After separation of hADSC-EVs from hADSCs condition medium by continuous centrifuge, the morphology was captured by TEM showing a lipid-bilayer vesicle with the diameter <200 nm (Fig. 2E). Figure 2F shows the representative image indicating that the phospholipid membrane probe can be successfully labeled to hADSC-EVs. As shown in Fig. 1G, the hADSC-EVs had a nano-scale size distribution with a largest average diameter of 190 nm according to NTA.

### Internalization of hADSC-EVs by osteoblast

To investigate whether hADSC-EVs could enter the cytoplasm of osteoblast, we incubated the labeled EVs with MG63 for 0.5, 4 and 8 h, respectively. Fluorescence microscopy images (Fig. 3A) showed that the hADSC-EVs labeled with PKH-26 (the red dots) were gradually internalized by the MG63 with the extension of incubation. At 0.5 h, nearly no hADSC-EVs distributed in the perinuclear region which indicated the EVs had not been internalized. At 8 h post-incubation, a large number of EVs have been internalized. The hADSC-EV uptake ratio (%) was 0, 54.27 ± 10.91 and 92.22 ± 7.52 at 0.5, 4 and 8 h, respectively (Fig. 3B).

### Observation of Ti and EV-Ti

Ti and EV-Ti were observed under fluorescent microscope and SEM to confirm the EVs immobilization and distribution. The red dot of labeled hADSC-EVs were detected and uniformly distributed on the surface of EV-Ti and there was no fluorescence observed in Ti group (Fig. 4A). SEM was used for further observation. The immobilization of hADSC-EVs on the surface of EV-Ti can be confirmed under low-magnification while there was nothing observed on the surface of Ti (Fig. 4B). Specifically, under high magnification, we observed that the immobilized EVs were gathering.

### Biocompatibility of EV-Ti *in vitro*

MG63 were seeded onto different materials and then F-actin were stained by phalloidin after incubated for 6 and 12 h, respectively, to evaluate cellular compatibility. At 6 h post-incubation, cells were sporadically distributed on the surface of each sample. And the MG63 cultured on EV-Ti showed regular spindle shaped morphology compared with that cultured on Ti which remained relatively round or with little spreading morphology (Fig. 5A). After 12 h of culture, cells were evenly distributed over the entire surface of EV-Ti substrate with well-organized actin filaments and obvious cellular pseudopod. While cells on the Ti surface still presented sporadic distribution and rounder spreading (Fig. 5B). The average cell project area (μm^2^) of Ti and EV-Ti group were 1002.68 ± 128.72 and 1632.38 ± 112.01 at 6 h and 1183.42 ± 229.15 and 1753.16 ± 11.79 at 12 h, respectively (Fig. 5C). The average cell perimeter (μm) of Ti and EV-Ti group were 165.55 ± 30.93 and 310.79 ± 44.56 at 6 h and 184.89 ± 40.32 and 361.01 ± 92.26 at 12 h, respectively, (Fig. 5D). The quantitative analysis indicated that the spreading area of osteoblast cultured on EV-Ti was larger than that cultured on Ti and the cells were well-spread on the EV-Ti after 6 h of culture.

### Osteoinduction of EV-Ti *in vitro*

To clarify whether EV-Ti could inductive osteo-differentiation, expression of osteo-related proteins and genes in MG63 cultured on Ti or EV-Ti for 14 days were measured (Fig. 6). As showing in immunofluorescence staining, the amount of COL 1 (Collagen 1, Fig. 6A) and OCN (Osteocalcin, Fig. 6B) produced by osteoblasts was visibly higher compared with Ti. The mean IOD of COL 1 were 74.7 ± 3.99 for Ti group, 199.69 ± 10.82 for EV-Ti group; the mean IOD of OCN were 96.31 ± 11.25 for Ti group and 257.2 ± 37.86 for EV-Ti group, respectively (Fig. 6C). As shown in Fig. 6D, expression of *ALP* (*Alkaline phosphatase*), *COL 1* and *OCN* mRNA on the EV-Ti group were upregulated as compared with the pristine Ti. Specifically, expression of *ALP*, *COL 1* and *OCN* were 1.51-, 1.68- and 1.82-fold in EV-Ti group than that in Ti group.

## Discussion

Ti is the most widely used clinical metallic implant material. However, the unsatisfied osseointegration caused by barren bioactivity on pristine Ti has limited its further application. Surface modification of Ti materials with bioactive components is an effective way to improve its biological activity and has been widely studied for decades [21]. Components with potential to be applied for improving bioactivity include bioceramics, ions and biomolecules. MSC-EVs are rich in multiple biomolecules and can be coated onto biomaterials to improve their bioactivity [15, 22]. Therefore, we chose ADSC-EV as bioactive component to improve the surface properties of Ti implant. The EV-Ti was fabricated by coating ADSC-EV onto fibronectin treated Ti implant. And the cellular compatibility and the osteoinductive ability were improved when compared with pure Ti implant (Fig. 1).

ADSCs were first discovered and defined as MSCs by Zuk *et al.* [23]. Human ADSCs, isolated from liposuction by classical collagenase digestion, used in this study occupied adipogenic, osteogenic and chondrogenic differentiation ability and expressed corresponding immunophenotype markers [24] (Fig. 2A−D). In recent years, ADSCs have appeared as a promising approach for various tissue regeneration. It was originally considered that the therapeutic effect of MSCs was attribute to their migration, engraftment and differentiation ability. Nevertheless, recent studies have indicated that they exert their therapeutic benefit mainly via the secretion of bioactive factors including numerous EVs [25, 26]. Based on their biogenesis and diameter, they can be classified into endosome-derived exosomes (30–150 nm), plasma membrane-derived microvesicles (100–1,000 nm) and apoptotic bodies (1,000–3,000 nm) [24]. The diameter distribution of collected ADSC-EVs in our study range from 80 to 828 nm and the peak diameter were 190 nm **(**Fig. 2G**)**, which may compose with exosomes and microvesicles. MSC-EVs have been demonstrated to affect recipient cell behavior and modify physiological process through transporting their bioactive cargos. Therefore, the cellular uptake process is critical to further application. We observed that osteoblast could internalize ADSC-EVs with 8 h at 37°C **(**Fig. 3**)**. The lasted study has proved that EVs enter cells predominantly via clathrin-independent endocytosis and micropinocytosis [27, 28].

With recent progress, many methods have been used to graft biocompatible molecules onto Ti implants such as heparin chemistry, chitosan functional coating, layer-by-layer method and so on [29, 31]. Fibronectin is a matrix protein that can be simply anchored onto Ti implant by passive physisorption. In addition, our previous research has discovered that fibronectin can be used to tether MSC-EVs to decalcified bone matrix [20]. Consequently, in the present study, fibronectin coating was adopted to adsorb ADSC-EVs onto Ti substrates. Florescence observation demonstrated that ADSC-EVs were evenly distributed onto the surface of material **(**Fig. 4A**)**. Additionally, EVs remained membrane integrity, which is critical for cell-to-cell communication, when adhered to scaffolds as revealed by SEM **(**Fig. 4B**)**.

To observe whether EV-Ti have suitable bioactivity, human osteoblast MG63 were cultured onto different substrates and their morphology and osteo-related gene expression were evaluated. Osteoblast cell line MG63 was derived from juxtacortical osteosarcoma and widely used to detect cell-related functions of bone repair material for their osteoblastic traits [32]. As shown in Fig. 5A and B, MG63 incubated on EV-Ti were well-expanding and exhibited larger spreading area than that on Ti, which indicated a healthy cell state. This may ADSCribe to MSC-EVs contained microRNAs and proteins responsible for actin cytoskeleton regulation [33, 34]. In addition, previous study has demonstrated that fibronectin coating onto Ti could favor MG63 adhesion, migration and proliferation [35]. The cell area and perimeter exhibited in Fig. 5C and D supported the qualitative results.

During osteo-differentiation, osteoblast will express several canonical markers including ALP, COL 1 and OCN. Specifically, ALP is an extracellular enzyme, which is highly expressed in activated osteoblast cell membrane; COL1 is a main structural protein of bone and its expression is significantly increased during osteogenic differentiation; and OCN is a marker of mature osteoblasts [36, 37]. We observed an increased expression of osteo-related genes and proteins mentioned above in MG63 seeded on EV-Ti, indicating a surface-dependent osteoinduction ability of EV-Ti **(**Fig. 6**)**. Interesting, new bone formation could not be observed in ectopic osteogenesis test (Supplementary data). The possible reason may be that the MG63 seeded on each substrate could not survive in harsh subcutaneous environment. Altogether, ADSC-EV grafting favored osteoblast adhesion and osteo-differentiation on fibronectin treated Ti.

MSC-EVs mediated cell-free therapy has received increasing attention. Compared with MSCs transplantation, their secreted EVs have multiple advantages including low risk of tumorigenicity, decreased immunogenicity and long-term preservation. And to our knowledge, large-scale production of clinical-grade EVs have been reported [38]. Combining this and previous studies, we have demonstrated that MSC-EVs can improve the biological activity of biomaterials by this simple coating method. This will provide an easy and effective way to modify the surface of biological materials.

## Supplementary data

Supplementary data are available at *REGBIO* online.

## Funding

This work was supported by the National Key R&D Program of China (2019YFA0110500), and the National Natural Science Foundation of China (No. 81873941, 81701922 and 81701912). 

*Conflict of interest statement*. None declared.

## Supplementary Material

rbaa038_Supplementary_Data
