# Supplementary material for: Simple application of adipose-derived stem cell-derived extracellular vesicles coating enhances cytocompatibility and osteoinductivity of titanium implant
Source: Regen Biomater. 2020 Dec 3;8(1):rbaa038. doi: 10.1093/rb/rbaa038 (PMC7947573; doi:10.1093/rb/rbaa038)
Supplement: rbaa038_Supplementary_Data [file rbaa038_supplementary_data.zip › suppl_data/Supplementary Data.docx]

Supplementary Materials for

**Simple Application of Adipose-Derived Stem Cell Derived Extracellular Vesicles Coating Enhances Cytocompatibility and Osteoinductivity of Titanium Implant**

**Lifeng Chen^a, b^, Shan Mou^a, b^, Jinfei Hou^a, b^, Huimin Fang^a, b^, Yuyang Zeng^a, b^, Jiaming Sun^a, b,^ *, Zhenxing Wang^a, b,^ ***

a Department of Plastic Surgery, Union Hospital, Tongji Medical College, Huazhong University of Science and Technology, Wuhan 430022, China.

b Wuhan Clinical Research Center for Superficial Organ Reconstruction, Wuhan 430022, China

* Corresponding authors

Corresponding authors’ e-mail addresses:

* Zhenxing Wang: wangzhenxing@hust.edu.cn

* Jiaming Sun: sunjm1592@sina.com

**This file includes:**

Methods for ectopic osteogenesis

Figure S1

Figure caption for Fig, S1

**Methods for ectopic osteogenesis**

Male BALB/c-nude mice (8 weeks old) were chose for experiment and purchased from Beijing Vital River Laboratory Animal Technology Co., Ltd. All procedures were performed according to the guidelines of the Ethics Committee of Huazhong University of Science and Technology.

Two different samples (Ti, and EV-Ti) were seeded with MG63 cells for 14 days. Then 10 mice were randomly divided into two groups (n=5 mice/group). After inhalation anesthesia (3% isoﬂurane), recipient mice were subcutaneously implanted with corresponding substrates. One month after implantation, the grafts were harvested for further immunohistochemical analysis (HE and OCN staining).

**Figure Caption for Fig, S1**

Ectopic osteo-inductive ability of EV-Ti in vivo. (A) Illustration of the experimental procedure. Representative HE images (B) indicating that there were no significant calcium nodules formed in both two groups. And immunohistochemical staining (C) showed OCN-positive cells may not be obvious in both tissues.

**Figure. S1**

**
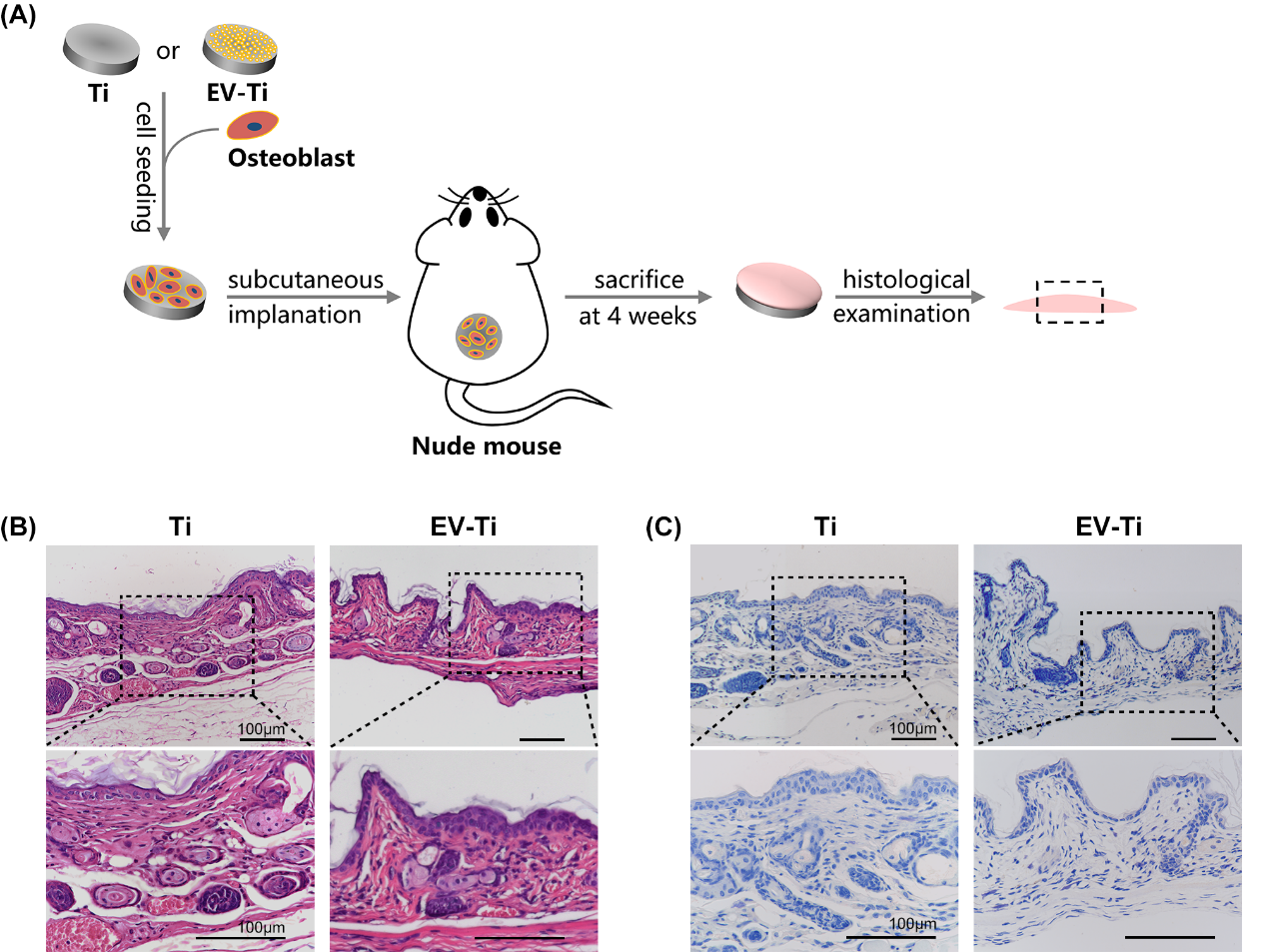
**
