# Supplementary material for: Simple application of adipose-derived stem cell-derived extracellular vesicles coating enhances cytocompatibility and osteoinductivity of titanium implant
Source: Regen Biomater. 2020 Dec 3;8(1):rbaa038. doi: 10.1093/rb/rbaa038 (PMC7947573; doi:10.1093/rb/rbaa038)
Supplement: rbaa038_Supplementary_Data [file rbaa038_supplementary_data.zip › suppl_data/Responding letter (2).docx]

**Department of Plastic Surgery, Wuhan Union Hospital, Huazhong University of Science and Technology, 1277 Jiefang Avenue, Wuhan,430022, China.**

**Tel: +86-27-85726445; Fax: +86-27-85726445**

**Zhenxing Wang**

**M.D. & Ph.D.**

**Email: benjamin.wzx@163.com**

**Dr. Nicholas Peppas**

**Editor-in-Chief, Regenerative Biomaterials**

**Email:** [**peppas@che.utexas.edu**](mailto:peppas@che.utexas.edu)

**Jun 10, 2020**

**Dear Nicholas Peppas:**

**RE：** Revision of Manuscript entitled “Simple Application of Adipose-Derived Stem Cell Derived Extracellular Vesicles Coating Enhances Cytocompatibility and Osteoinductivity of Titanium Implant”

We would like to cordially appreciate your kind consideration of our manuscript. We have carefully read the reviewers` valuable comments and prepared our responses to the reviewers’ comments as follows.

**Response to reviewers**

**Reviewer #1:**

Nanoparticles on Ti surface is not well explained. SiC sandpaper cannot result in such a morphology. Please give data of EDS and higher magnification. Without a reasonable explain, the authenticity of the data is doubtful.

**Response:**

It is very kind for your rigorous comment. As your request, the higher magnification of titanium implant used in this manuscript was observed and corresponding EDS data was collected. As shown in Figure 1, under low magnification (10000× or 80000×), both groups demonstrate similar morphology. However, while scanning under high magnification (30 000×), the SEM images present different morphology. Nanoparticle morphology was obviously observed on polished substrate compared to that on unpolished titanium. Moreover, EDS surface and point scanning showed that both samples were composited with Ti element. Otherwise, in order to observe nanoscale hADSC-EV, the SEM images used in our manuscript were captured by high resolution equipment (Hitachi SU8100), which could probably obtain more details. Altogether, we believe that the nanoparticle morphology might inherent feature which could be observed under high magnification.

Figure 1. SEM observation and EDS data of titanium implant. A, SEM images of unpolished and polished titanium substrates. B, EDS surface scanning (under 10000× magnification) and point scanning (red point in figure 1.A) of titanium implant.

**Reviewer #2:**

The manuscript is currently a self-contained presentation. The figures and captions are clear and relevant. I thus recommend its acceptance in the high quality journal Regenerative Biomaterials.

**Response:**

Thanks for your kind affirmation. I’m looking forward to my manuscript being accepted by the high quality journal Regenerative Biomaterials.
