# Supplementary material for: Simple application of adipose-derived stem cell-derived extracellular vesicles coating enhances cytocompatibility and osteoinductivity of titanium implant
Source: Regen Biomater. 2020 Dec 3;8(1):rbaa038. doi: 10.1093/rb/rbaa038 (PMC7947573; doi:10.1093/rb/rbaa038)
Supplement: rbaa038_Supplementary_Data [file rbaa038_supplementary_data.zip › suppl_data/Responding letter.docx]

**Department of Plastic Surgery, Wuhan Union Hospital, Huazhong University of Science and Technology, 1277 Jiefang Avenue, Wuhan,430022, China.**

**Tel: +86-27-85726445; Fax: +86-27-85726445**

**Zhenxing Wang**

**M.D. & Ph.D.**

**Email: benjamin.wzx@163.com**

**Dr. Nicholas Peppas**

**Editor-in-Chief, Regenerative Biomaterials**

**Email:** [**peppas@che.utexas.edu**](mailto:peppas@che.utexas.edu)

**Jun 10, 2020**

**Dear Nicholas Peppas:**

**RE：** Revision of Manuscript entitled “Simple Application of Adipose-Derived Stem Cell Derived Extracellular Vesicles Coating Enhances Cytocompatibility and Osteoinductivity of Titanium Implant”

We would like to cordially appreciate your kind consideration of our manuscript. We have carefully read the reviewer’s valuable comments and prepared our responses to the reviewer’s comments as follows.

**Response to reviewers**

**Reviewer: 2**

Comments to the Author
The author still didn't answer the question that why nanostructures appeared on the polished titanium. This is an interesting finding which is very different from previous report. The author should give a reasonable explanation.

**Response:**

We are apologized for our last ambiguous responding letter. We believe there are two reasons for why nanostructures appeared on the polished titanium.

Firstly, the process of polish may result in significant nanoparticle morphology onto titanium implant. As shown in Fig 1.C and Fig 1.F, under high magnification (300 000×), nanoparticle morphology could be observed both on unpolished and polished titanium. Furthermore, the nanostructure was obvious onto polished substrate compared to that onto unpolished substrate.

Secondly, the nanoparticle structure could be observed might because we used high SEM magnification field in present research. In order to observe extracellular vesicles on the implant, we used a high magnification that isn’t commonly used in other researches. As shown in Fig 1.A, Fig 1.B, Fig 1.D and Fig 1.E, under low magnification (10 000× or 80 000×), both groups demonstrate similar morphology. However, while scanning under high magnification (300 000×), the SEM images present more detailed morphology which is different under low magnification. Moreover, under low magnification (10 000× or 80 000×), the morphology of titanium in our study were similar compared to other research (Fig 2, CON group). The high magnification in our study present more details which could not be observed on low magnification.

Altogether, we believe that the nanostructures may be resulted from polish process and the high magnified SEM observation make them significantly.

Figure 1. SEM observation and EDS data of titanium implant. A, SEM images of unpolished and polished titanium substrates. B, EDS surface scanning (under 10000× magnification) and point scanning (red point in figure 1.A) of titanium implant.

Figure 2. Images of titanium implants cited from “Liang J, Xu S, Shen M, Cheng B, Li Y, Liu X, Qin D, Bellare A, Kong L. Osteogenic activity of titanium surfaces with hierarchical micro-/nano-structures obtained by hydrofluoric acid treatment[J]. Int J Nanomedicine, 2017,12:1317-1328.”
